# Cases Surgical and Obstetrical

**Published:** 1845-11

**Authors:** J. Emery

**Affiliations:** Woodville, Missouri


Art. VII.—*Cases Surgical and Obstetrical.* By Dr. J. Emery, of Woodville, Mo.

Case I.—On the 13th of August I was called to see a child aged two years, the son of Mrs. B. From all the attending symptoms I directly concluded that some foreign body had gained admittance into the trachea. After trying various means for its removal without success, I determined to perform the operation of tracheotomy, which I did with the assistance of my partner, E. E. Hand. I made an incision along the median line, about two inches in length; in the course of which I divided two veins, but easily arrested the hemorrhage by pressure; I next opened the trachea to the extent of five rings, immediately upon which a considerable quantity of mucus, together with several small pieces of corn were discharged.

The child was now perfectly motionless, its voice hushed, and its pulse so low as to be scarcely perceptible. I now had its body elevated considerably above its head, and the trachea held open with blunt hooks; at the same time I introduced a probe into the right bronchus, which caused a grain of corn to be expelled with a strong gust of a air. I then closed the wound with stitches, adhesive plaster, &c.; with the exception of a small opening in the upper portion of the incision, which was left unclosed in consequence of the patient's laborious breathing. This opening I did not close for several days, (fearing suffocation,) the child breathing during the time partially through it. There was but little secondary fever, venesection being required but once.

The wound is now (August 30th) entirely closed, and the child nearly well.

I do not report this case on account of its originality, but to encourage others to perform the operation when required, as I have known several cases in which, I believe, life has been lost for want of it.

Case II.—On the 26th of June, I was requested to visit Mrs. M., aged about 25 years, in the sixth month of pregnancy with her third child. By a sudden lift symptoms of abortion had been brought on, in which state she had been several days, at the time of my visit. During this time she was under the treatment of a steam quack, who had vomited and steamed her, I suppose, to his entire satisfaction.

I found her with a high fever; tongue dry and brown; bowels costive; the os uteri dilated; and with powerful uterine contractions. I bled her freely, gave camphor and opium, together with a gentle laxative. When I left her in the evening, she was quite comfortable. Next morning was called in haste to see her. I found that the uterine contractions had returned with increased violence, the os uteri was dilated, and the membranes protruded nearly as large as a hen's egg.

I now gave up all hope of preventing abortion. After waiting some time, the pains being rather tardy, I determined to give ergot, and accordingly prepared a drachm in decoction; I gave one-third of this every twenty minutes. But contrary to my expectations, under the influence of the ergot, the membranes receded, and the os uteri gradually closed. The patient went her full time, and was delivered of a healthy child.

I make no remarks on the above case, but simply state the facts as they occurred. Has any one else observed similar effects from the exhibition of ergot?

September 6th, 1845.

